# Effects of Gender and Age on Dietary Intake and Body Mass Index in Hypertensive Patients: Analysis of the Korea National Health and Nutrition Examination

**DOI:** 10.3390/ijerph17124482

**Published:** 2020-06-22

**Authors:** Hyunju Dan, Jiyoung Kim, Oksoo Kim

**Affiliations:** 1 College of Nursing, Ewha Womans University, Seoul 03760, Korea; hidan@hanmail.net; 2 Department of Nursing, Sangmyung University, Cheonan-si 31066, Korea; jy1223kim@smu.ac.kr

**Keywords:** middle aged, aged, hypertension, nutrients, body weight maintenance

## Abstract

Controlling weight and dietary intake are important for hypertensive patients to manage their blood pressure. However, the interaction effect of gender and age on weight and dietary intake is not well known. The aim of this study was to examine the main and interaction effects of age and gender on body mass index (BMI) and dietary intake in hypertensive patients. We analyzed data from 4287 participants with hypertension (1600 participants 45–64 years old and 2687 participants 65 years or older) who participated in the Korea National Health and Nutrition Examination Survey (2013–2016). Two-way ANOVAs were conducted to examine the main and interaction effects of age and gender on BMI and dietary intake. Gender and age had significant main effects on BMI, intake of energy, cholesterol, sodium, and potassium. However, both gender and age illustrated interaction effects on BMI (F = 8.398, *p* = 0.004), energy intake (F = 12.882, *p* < 0.001), and cholesterol intake (F = 6.107, *p* = 0.014), while not showing any significant interaction effects on sodium (F = 3.547, *p* = 0.060) and potassium (F = 3.396, *p* = 0.066). Compared to the middle-aged group, BMI, energy intake, and cholesterol intake decreased in the older-aged group. However, the declines were steeper in men than in women. Therefore, both gender and age need to be considered for weight and dietary intake management for hypertensive patients.

## 1. Introduction

Hypertension is one of the most common chronic diseases and is a major risk factor for cardiovascular disease (CVD) and stroke. Uncontrolled hypertension is associated with CVD mortality and premature vascular death [1,2,3]. The prevalence of hypertension in Korea in 2016 was 35.0% for men and 22.9% for women [4]. The overall prevalence of hypertension among US adults was 32.9% for men and 27.0% for women in 2017 [5]. The management and treatment of hypertension among middle-aged and older-aged people are essential, as hypertension increases in frequency and risk of mortality with age [3].

Age, gender, energy and nutrient intake, blood lipids, and high body mass index (BMI) have been reported to be associated with hypertension prevalence [6,7,8]. High blood lipids levels result in atherosclerosis of the blood vessels, leading to hypertension or exacerbation of hypertension [9]. Obesity is a major risk factor for hypertension, because it activates the renin-angiotensin system [10]. Therefore, effective management should consider factors associated with hypertension.

Lifestyle modification is effective in lowering blood pressure, and should be applied to all hypertensive people [11]. Hypertension treatment can be divided into medicinal and non-medicinal (e.g., life-style changes) treatments. The Eighth Joint National Committee on Prevention, Detection, Evaluation, and Treatment of High Blood Pressure (JNC-8) provides guidelines for hypertension management, such as dietary approaches to stop hypertension (DASH), weight-loss focused diets, and the reduction of daily sodium intake to <2400 mg [12]. These diets were found to be effective for preventing chronic diseases by improving blood indicators, including cholesterol and glucose [13].

In particular, the long-term over-consumption of sodium has been found to increase the incidence of hypertension, stroke, heart disease, kidney disease, and stomach cancer [14]. Koreans ingest many salty foods, like kimchi, salted fish, and soup, so adopting a low-sodium diet for blood-pressure control is difficult for many people [15]. Dietary potassium intake has been demonstrated to lower blood pressure in hypertensive patients [16]. However, Lee, Lee, Ko, and Ly [17] reported that there was no significant relationship between potassium intake and blood pressure in Korean men, while systolic blood pressure was high when potassium intake was low in middle-aged women. The Korean government recommends increasing potassium intake to reduce sodium absorption, because of the population’s high sodium intake [18].

In a large prospective study, dietary habits varied according to the gender of middle-aged and older-aged Koreans, with men preferring a traditional diet and women preferring a Western-style diet [19]. Ok, Ko, and Ryu [20] found that among hypertensive people, women and older people were more likely to maintain healthy behaviors, including dietary habits, which indicates that dietary habits vary according to age and gender. Therefore, it is necessary to analyze dietary intake and BMI according to gender and age. The purpose of this study was to examine the main and interaction effects of gender and age on BMI and dietary intake of hypertensive patients using data from the Korean National Health and Nutrition Examination Survey (KNHANES).

## 2. Materials and Methods

### 2.1. Setting and Participants

This study used data from the sixth (2013–2015) and the first year of the seventh (2016) KNHANES, conducted by the Korean Centers for Disease Control and Prevention. The KNHANES has been conducted since 1998 to identify the status and trends of health and nutrition of the population, and it has been carried out annually since 2007 on a three-year basis. Participants were extracted using the 2010 Population and Housing Census data via a multi-level stratified cluster sampling method based on residential area, gender, age, and housing type. The KNHANES data were collected from a health interview survey, a health examination survey, and a nutrition survey. We analyzed data from 1600 middle-aged patients with hypertension (45–64 years) and 2687 older-aged patients with hypertension (65 years or older) in the KNHANES dataset. In the middle-aged group, there were 721 men and 879 women. The older-aged group consists of 1066 men and 1621 women.

### 2.2. Measures

#### 2.2.1. Body Mass Index (BMI)

Body weight and height were measured by trained investigators. BMI was calculated by dividing patient weight (kg) by square of height (m^2^). BMI was categorized as underweight (<18.5 kg/m^2^), normal (18.5–22.9 kg/m^2^), overweight (23–24.9 kg/m^2^), or obese (≥25 kg/m^2^), under the criteria from the World Health Organization’s Asia-Pacific region and the Korean Society for the Study of Obesity [21].

#### 2.2.2. Dietary Intake

Dietary intake was determined by examining all foods consumed during the day through individual interviews. In this study, four dietary components related to hypertension, comprising energy, cholesterol, sodium, and potassium, were used for analysis.

### 2.3. Ethical Considerations

The KNHANES was reviewed by the Institutional Review Board of the Korean Centers for Disease Control and Prevention (IRB No.: 2013-07CON-03-4C, 2013-12EXP-03-5C, 2015-01-02-6C), and we officially requested use of and downloaded the data from its website (http://knhanes.cdc.go.kr), which discloses the KNHANES raw data to the public. The study received confirmation of exemption from deliberation by the Ewha Womans University’s Ethics Review Committee (IRB No.: ewha-201810-0005-01).

### 2.4. Data Analysis

Statistical analyses were performed using a complex sampling design analysis module in SPSS version 21 (IBM, Chicago, IL, USA). In this study, data from the sixth (2013–2015) and the first year of the seventh (2016) KNHANES were integrated by applying weighting, stratification variables, and cluster variables for complex sample analysis to maintain the population representativeness of the samples. The variable frequency was assessed for unweighted frequency. A frequency analysis of complex samples was conducted to identify the general characteristics of middle-aged and older-aged people with hypertension, such as income, education, and marital status.

To determine BMI level and dietary intake, a complex-sample descriptive-statistics analysis was conducted to estimate means and standard errors. Differences by gender and age in BMI were analyzed using the Rao-Scott chi-square test, a modified form of the Pearson chi-square test that processes complex sampling designs [22]. A two-way ANOVA was performed, to determine the differences in BMI and dietary intake by gender and age (middle-aged/older-aged).

## 3. Results

### 3.1. Participant Characteristics

From 2013 to 2016, 31,098 people participated in the KNHANES, of which 15,013 were 45 years or older. Our final analyses for this study comprised 4287 hypertensive participants aged 45 years or older who accounted for 28.6% of the total the KNHANES participants older than 45. Descriptive statistics for age, income, education, marital status, and BMI by age and gender are presented in Table 1.

### 3.2. BMI by Gender and Age

BMI by gender and age group are presented in Table 1. Among the middle-aged group, 393 (57.8%) were obese and 177 (23.4%) were overweight in men and 475 (53.3%) were obese and 223 (24.2%) were overweight in women. Among the older-aged group, 427 (39.4%) were obese and 288 (28.5%) were overweight in men and 755 (47.44%) were obese and 422 (26.0%) were overweight in women.

### 3.3. BMI and Dietary Intake by Gender, Age, and Gender-Age Interaction

**Figure 1:**
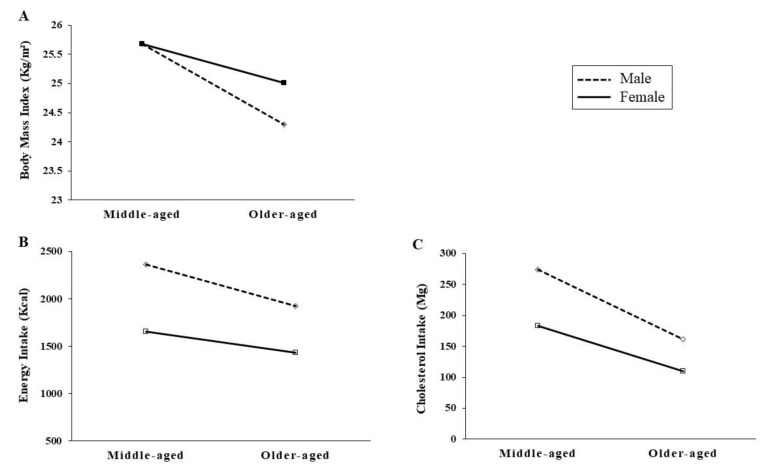
Interaction effects of gender and age on BMI (**A**), energy (**B**) and cholesterol intake (**C**).

The main and interaction effects by gender and age on BMI and dietary intake are shown in Table 2 and Table 3. Our results indicate that BMI differed significantly by gender (F = 8.524, *p* = 0.004) and age (F = 63.628, *p* < 0.001), and that the gender-age interaction was also significant (F = 8.398, *p* = 0.004) (Figure 1).

Two-way ANOVAs for gender and age group were performed, to investigate the main and interaction effects on dietary intake, including energy, cholesterol, sodium, and potassium intake (Table 3). Gender had significant main effects on energy (F = 420.282, *p* < 0.001) and cholesterol intake (F = 86.604, *p* < 0.001). Age group (middle-aged versus older-aged) had main effects on energy (F = 129.717, *p* < 0.001) and cholesterol intake (F = 137.678, *p* < 0.001) (Figure 1).

Sodium intake was lower in the older age group than in the middle-aged group (F = 37.702, *p* < 0.001) and was lower in women than in men (F = 248.211, *p* < 0.001). Potassium intake showed a similar pattern to sodium intake, where age (F = 78.868, *p* < 0.001) and gender (F = 103.907, *p* < 0.001) had both significant main effects on potassium intake, but there were no significant interaction effects on sodium (F = 3.547, *p* = 0.060) and potassium (F = 3.396, *p* = 0.066) (Table 3).

## 4. Discussion

This study was conducted to examine the main and interaction effects of age and gender on dietary intake and BMI for hypertensive patients from the KNHANES dataset. We found that age and gender had the main and interaction effects on BMI, energy and cholesterol intake among patients with hypertension.

Age and gender had interaction effects on BMI and energy intake in this study. There was no difference in mean BMI between men and women who were middle-aged, but older-aged men had a lower BMI than older women. However, among older-aged female patients, energy intake was below the estimated energy requirement (EER) of 1600 kcal/day [18]. Most hypertensive patients in this study exceeded the normal BMI range, according to the WHO Asia-Pacific guidelines. BMI reflects an individual’s eating attitudes as related to their physical fitness [23]. Obesity in older adults has been associated with physical activity and mobility [24]. In a study of people over 60 years old in Sweden, the BMI of older women was higher than that of older men. Additionally, older women were more likely to walk less and perform fewer outdoor activities than men [25]. Additionally, to avoid/reduce obesity, proper exercise should be performed. Obesity is associated with the development of hypertension and depression [26]. Proper weight maintenance is important, not only to prevent hypertension onset, but also to promote better health among hypertensive patients. Therefore, interventions to maintain normal weight in hypertensive patients after middle age should be established.

Compared to the middle-aged group, cholesterol intake decreased in the older-aged group. Men showed steeper declines than women did in this study. The standard for cholesterol intake in Koreans of both genders over 19 years old is <300 mg/day [18]. In this study, the cholesterol intake of older-aged women was 109 mg/day, which is lower than the recommended standard. Due to lack of evidence that dietary cholesterol affects blood cholesterol, Dietary Guidelines for Americans 2015–2020 [27] removed the recommendation for limiting cholesterol intake to below 300 mg per day, and limiting egg intake to two or fewer per day. High protein foods are needed to prevent muscle loss from aging. Eggs contain a high level of cholesterol but are low in saturated fat, and they are one of the major sources of protein [27]. In addition, HDL in cholesterol promotes breakdown of beta amyloid, a dementia risk factor, to help prevent dementia [28,29], and has been shown to play an important role in preventing cardiovascular diseases [28]. Therefore, low cholesterol levels in older-aged women with high blood pressure can adversely affect muscle and cardiovascular health, so it is necessary to maintain proper cholesterol levels through diet.

Age and gender had main effects on sodium and potassium intake, but illustrated not significant interaction effects on both sodium and potassium intake. Sodium intake of the participants in this study exceeded the target of 2000 mg/day [18] for both age groups and genders. Dietary Guidelines for Americans 2015–2020 [27] states a limit of 2300 mg of sodium per day. However, the middle-aged men in this study, despite being hypertensive, showed consumption level twice as high as the Korean and US standards. The average sodium intake of Koreans is 3255 mg/day, which is higher than the recommended amount. However, male participants in this study consumed more sodium than the Korean average [30]. A study of middle-aged Korean workers found that higher sodium intake was associated with more frequent dining out [21]. Middle-aged Korean men often eat outside the home with colleagues for work-related events, and typically eat meat while drinking alcohol, which is associated with high sodium intake. Therefore, to reduce the sodium intake of middle-aged men in Korea, continuous education is needed at the workplace and government levels. In addition, smoking and alcohol intake in Korean men have been associated with increased sodium intake [31]. Since excessive sodium intake in middle-aged men is associated with an unhealthy lifestyle, sodium intake should be managed in parallel with healthy lifestyle practices.

This study shows that patients with hypertension consume about twice the target sodium intake of the general population, even though hypertensive patients should have more restricted salt intake than the general population. In Korea, the kimchi (traditional Korean side dish made from salted and fermented Napa cabbage) helps high vegetable intake, and high levels of vegetable intake have been reported to reduce blood pressure [32]. However, at the same time, the kimchi consumption is also associated with elevated sodium intake [17]. Most Koreans eat kimchi and salty stew daily. In other words, excessive sodium intake in Koreans is closely tied to traditional eating habits. Therefore, health care providers need to provide hypertension patients with information on cooking methods that use less salt. Older-aged women have lower potassium intake (3500 mg/day) than older-aged men and people of all other age groups in Korea [18]. Potassium supplementation is recommended [33], because it reduces elevated blood pressure from the excessive salt intake. A meta-analysis study showed that consuming 3500–4700 mg/day of potassium lowered systolic blood pressure by 7.16 mmHg and diastolic blood pressure by 4.01 mmHg [34]. For Koreans, foods high in potassium comprise traditional meals, mainly rice and vegetables [17]. Therefore, older-aged women should be encouraged to consume enough potassium based on the traditional Korean diet. Education about sufficient potassium intake and a low-sodium diet should be provided to hypertensive patients in the middle- and older-aged groups of both genders to improve sodium-absorption reduction.

It has been reported that hypertensive patients who fail to comply with healthy dietary behavior may be due to insufficient information on dietary guidelines or individual differences in diet preferences [35]. The Mediterranean diet is known to reduce the risk of hypertension [36]. Therefore, individually tailored dietary education should be provided to hypertensive patients, to improve their understanding of dietary management, and to promote healthier habits.

This study has several limitations. Since this study used a cross-sectional study design, there is a limit to explaining the causal relationship. Nevertheless, it is meaningful to analyze BMI and dietary intake of hypertensive patients based on national data. In this study, a recall bias should be considered, because the dietary intake was not measured objectively, but self-reported. Socioeconomic variables and physical activities are also considered to affect dietary intake. However, in this study, physical activities and socioeconomic factors, such as income level and living arrangements that may affect dietary intake were not considered.

This study found that BMI, calorie intake, and cholesterol intake in hypertensive patients were influenced by both gender and age. In this study, sodium intake was high, even in hypertensive patients. In the middle-aged men, sodium intake was twice the recommended amount. Therefore, to control the blood pressure of hypertensive patients, health care providers need to educate patients to reduce sodium intake. Gender and age should be considered when aiming to control the nutrient intake and weight for hypertension management.

## 5. Conclusions

In this study, age and gender had main and interaction effects on BMI, energy, and cholesterol intake in hypertensive patients. Compared to the middle-aged group, BMI, energy intake, and cholesterol intake decreased in the older-aged group. However, the decline of BMI and energy intake and cholesterol intake were steeper in men than in women. Our findings suggest that an age- and gender-specific approach is critical for improving the dietary habits of hypertensive patients.

## Figures and Tables

**Table 1:** General characteristics of the participants (N = 4287).

Variables	Middle-Aged		Older-Aged	
	Men	Women	Men	Women
	N (%)	N (%)	N (%)	N (%)
Age (Mean ± SE)	55.25 ± 0.233	56.45 ± 0.202	72.44 ± 0.173	73.30 ± 0.139
Income level quartile				
Highest	187 (25.8)	191 (22.8)	263 (27.7)	397 (26.9)
Medium highest	168 (22.7)	188 (22.3)	261 (23.6)	406 (23.5)
Medium lowest	168 (23.8)	265 (26.4)	277 (23.7)	425 (25.4)
Lowest	196 (27.7)	265 (28.5)	256 (24.9)	385 (24.3)
Education				
Elementary school or less	128 (15.5)	334 (34.5)	436 (42.3)	1234 (78.4)
Middle school	116 (14.3)	181 (22.2)	178 (16.9)	153 (9.9)
High school	253 (38.3)	248 (32.0)	280 (27.1)	131 (8.9)
University or more	204 (31.9)	96 (11.3)	140 (13.7)	43 (2.7)
Marital status				
Married	629 (86.9)	687 (79.7)	923 (87.3)	733 (42.7)
Separated/divorce	57 (7.3)	74 (8.5)	51 (5.1)	54 (3.5)
Widowed	8 (1.2)	109 (11.1)	85 (7.3)	823 (53.1)
Unmarried	27(4.6)	9 (0.7)	4 (0.3)	11 (0.7)
BMI				
Underweight (<18.5 kg/m^2^)	7 (0.8)	4 (0.5)	18 (2.0)	26 (1.7)
Normal (18.5–22.9 kg/m^2^)	138 (18.0)	177 (22.1)	331 (30.0)	414 (24.9)
Overweight (23–24.9 kg/m^2^)	177 (23.4)	223 (24.2)	288 (28.5)	422 (26.0)
Obese (≥25 kg/m^2^)	393 (57.8)	475 (53.3)	427 (39.4)	755 (47.4)

BMI, body mass index.

**Table 2:** Body mass index (BMI) and dietary intake by gender and age (N = 4287).

Variables	Middle-Aged		Older-Aged	
	Men	Women	Men	Women
	Mean ± SE	Mean ± SE	Mean ± SE	Mean ± SE
BMI (kg/m^2^)	25.68 ± 0.149	25.68 ± 0.145	24.30 ± 0.101	25.01 ± 0.096
Energy (Kcal/day)	2364.69 ± 44.461	1659.01 ± 24.350	1927.73 ± 26.646	1432.96 ± 17.725
Cholesterol (mg/day)	274.27 ± 11.438	183.68 ± 7.289	161.54 ± 6.779	109.46 ± 4.501
Sodium (mg/day)	4526.92 ± 135.087	2968.18 ± 68.289	3770.40 ± 93.738	2565.80 ± 53.921
Potassium (mg/day)	3357.92 ± 68.252	2922.80 ± 63.062	2960.06 ± 57.530	2313.99 ± 39.525

BMI, body mass index.

**Table 3:** Main and interaction effects of gender and age on BMI and dietary intake (N = 4287).

Variables	Age Group		Gender		Age by Gender Interaction	
	F	*p*	F	*p*	F	*p*
BMI (kg/m^2^)	63.628	<0.001	8.524	0.004	8.398	0.004
Energy (Kcal/day)	129.717	<0.001	420.282	<0.001	12.882	<0.001
Cholesterol (mg/day)	137.678	<0.001	86.604	<0.001	6.107	0.014
Sodium (mg/day)	37.702	<0.001	248.211	<0.001	3.547	0.060
Potassium (mg/day)	78.868	<0.001	103.907	<0.001	3.396	0.066
